# N-rGO/S@porous SiC Composite with Multidimensional Hybrid Architectures for Structural Energy-Storing Applications

**DOI:** 10.3390/nano16110656

**Published:** 2026-05-23

**Authors:** Shasha Xiao, Xiaojia Li, Xiaojiang He, Lei Yuan, Xudong Liu

**Affiliations:** 1 Laser Fusion Research Center, China Academy of Engineering Physics, Mianyang 621900, China; xiaoshasha22@gscaep.ac.cn (S.X.); shakalee@pku.edu.cn (X.L.); hexj@mail.ustc.edu.cn (X.H.); yuanlei0211@163.com (L.Y.); 2 School of Chemistry and Materials Science, University of Science and Technology of China, Hefei 230026, China

**Keywords:** structural energy storage, hydrothermal synthesis, graphene oxide, silicon carbide, supercapacitor

## Abstract

Currently, dual-functional composites that simultaneously provide structural support and energy storage capabilities have garnered significant attention. However, the challenge of balancing mechanical strength and energy storage performance remains a limiting factor for their application. Herein, a novel N-doped reduced graphene oxide/nano-sulfur@porous SiC (N-rGO/S@porous SiC) composite material was successfully prepared by in situ embedding N-rGO supported with nano-sulfur into a 3D-printed porous SiC scaffold via a hydrothermal synthesis approach. The hierarchical porous structure composed of SiC and N-rGO facilitates mass transport of the liquid electrolyte. Benefiting from the high strength of SiC, the novel material achieves a compressive strength of 93.5 MPa. Benefiting from the synergistic effect of the N-rGO/S composite and the high ionic conductivity of the liquid electrolyte, the electrode material delivers superior electrochemical energy storage performance, achieving a specific capacitance of 800.7 mF/cm^2^ at a current density of 1 mA/cm^2^, together with remarkable rate capability and good cycling stability. To our knowledge, this composite exhibits a high level of integrated properties. More importantly, the strategy of integrating porous, high-strength supports with high-performance electrode materials opens new avenues for the synthesis of structure-energy-storage dual-functional composites.

## 1. Introduction

Driven by the surging global energy demand and the rapid proliferation of electric vehicles, conventional energy systems are encountering pressing challenges associated with resource depletion. Accelerating the transition toward clean, efficient, and sustainable energy structures has become an urgent imperative, while research on high-performance energy storage devices has emerged as a central focus in the contemporary energy sector. Through the deep integration of energy storage units within structural carriers, spatial coupling and functional synergy between energy storage and load-bearing functions are realized. Such integration significantly reduces system redundancy, improves gravimetric energy density, and enables lightweight, integrated energy solutions for distributed generation, electric mobility, and aerospace applications. However, conventional structural-energy storage composites generally face an inherent trade-off between mechanical strength and electrochemical performance of electrode materials and electrolytes, limiting their widespread deployment in next-generation energy systems [1,2].

To address the trade-off, researchers have sought to integrate energy storage functionality into high-strength materials. Numerous studies have leveraged high-strength carbon fibers as structural scaffolds to explore this strategy. Shirshova et al. demonstrated supercapacitors with a specific capacitance of 52 mF/g, coupled with a compressive modulus of 12.5 GPa and strength of 7.5 MPa [3]. Qian et al. reported carbon aerogel-coated carbon fiber supercapacitors delivering 71 mF/g and a shear strength of 8.7 MPa [4]. Javaid et al. developed graphene nanosheet–carbon fiber composites, achieving 118.7 mF/cm^3^ volumetric capacitance and 2.78 GPa shear modulus [5]. Xu et al. attained 354 mF/g specific capacitance and 6.7 MPa shear strength via co-coating graphene nanosheets and carbon aerogel onto carbon fibers [6]. While these efforts confirm the dual functionality of carbon fiber-based systems, activation-induced porosity compromises mechanical integrity. The coating process is further impeded by the curvature of fibers, thereby limiting active material loading and giving rise to relatively low specific capacitance. Moreover, the intrinsic absence of electrolyte percolation networks in woven architectures mandates solid-state electrolytes, aggravating the mechanical–electrochemical imbalance.

Reduced graphene oxide (rGO) is a two-dimensional carbon material with interlayer oxygen-containing groups and chemically tailorable structures. It possesses a theoretical specific capacitance of up to 550 F/g, and experimentally achieved values typically range from 200 to 250 F/g, rendering it a promising electrode material for supercapacitors [7,8]. Self-standing rGO paper can act as both active material and current collector, realizing current collector-free supercapacitors with low cost, environmental friendliness, and good flexibility [9]. Individual graphene oxide monolayers exhibit high mechanical strength, yet the interlayer adhesion after film formation is poor, resulting in a tensile strength of only approximately 3~5 MPa [10,11,12]. To address these limitations, researchers have incorporated graphene oxide with polymeric binders, leveraging 3D printing to construct hierarchically porous three-dimensional architectures. The resultant high specific surface area and oriented pore channels facilitate mass transport and enhance ion transfer kinetics. The binder additionally reinforces interlayer adhesion between stacked graphene sheets, enabling tailored fabrication of high-areal-capacity supercapacitor electrodes. Relevant studies have fabricated high-performance 3D-printed graphene-based electrodes via direct writing and extrusion printing strategies to acquire outstanding areal capacitive properties. However, existing graphene assembly and 3D printing techniques are incapable of effectively improving mechanical performance. Even optimized structural designs can hardly achieve satisfactory mechanical strength [13,14,15]. Consequently, graphene-based materials (hundreds of kPa) remain orders of magnitude weaker than structural materials (several MPa), presenting a critical barrier to structural energy storage applications [16].

To address these challenges, this study integrates the lightweight, high-strength properties of SiC with the exceptional energy storage capacity of graphene oxide. A novel N-doped reduced graphene oxide/nano-sulfur@porous SiC (N-rGO/S@SiC) multidimensional composite was synthesized by a two-step strategy combining 3D printing and hydrothermal treatment. The porous SiC scaffold inherently provides ultra-high mechanical strength, while its porous architecture accommodates electrode materials and facilitates mass transport. Graphene serves dual functions: surface-coated graphene ensures high electrical conductivity, whereas pore-confined graphene delivers superior electrochemical energy storage. Nitrogen doping introduces additional pseudocapacitive contributions, while sulfur incorporation promotes secondary porosity development and reinforces interlayer bonding. These synergistic effects enhance specific capacitance and electrochemical stability concurrently. The resultant composite simultaneously exhibits exceptional mechanical and electrochemical performance, with a high areal specific capacitance of 800.7 mF/cm^2^, outstanding cycling stability (maintaining 92.5% of its initial capacitance after 5000 cycles), and an ultra-high compressive strength of 93.5 MPa. We anticipate that this three-dimensional structural-energy-storage dual-functional architecture strategy not only provides a feasible route for the design of advanced multifunctional energy storage materials but also can be extended to structural batteries and structural lithium–sulfur batteries, offering new insights for the development of integrated energy storage and structural systems.

## 2. Experiment

### 2.1. Materials

Polysiloxane resin (MK), Tetrahydrofuran (THF), Trimethylolpropane trimethacrylate (TMPTA), Isobornyl methacrylate (IBOMA), Tripropylene glycol monomethyl ether (TPM), 3-(Methacryloyloxy)propyltrimethoxysilane (MPS), 1-Hydroxycyclohexyl phenyl ketone (184, 1.5 wt%), 2,4,6-Trimethylbenzoylphosphinic acid ethyl ester (TPO-L, 1.5 wt%), HCl (1 mol/L), Graphite oxide (GO), Sulfur (S), NH_4_OH, DI water.

### 2.2. Material Synthesis

#### 2.2.1. Synthesis of Porous Sic Scaffold

Initially, 120 g of PMK was thoroughly dissolved in 120 mL of THF. Concentrated HCl (400 μL) and ultrapure water (600 μL) were added, and the mixture was hydrolyzed for 0.5 h. The hydrolysis and condensation mechanism is illustrated in Figure S1. Then, 15 g TMPTA, 30 g IBOMA, and 30 g TPM were added, and the mixture was stirred for an additional 0.5 h. MPS was added, and the mixture was sealed with aluminum foil and aged overnight (~12 h). The solvent was removed via rotary evaporation at 45 °C. Photoinitiators 184 (5‰ by mass) and TPO-L (3% wt%) were then added to the resulting resin under vigorous stirring to yield a photopolymerizable polysiloxane paste. The paste was patterned into predesigned SiC scaffold architectures via digital light processing (DLP) 3D printing, followed by high-temperature sintering to obtain the final SiC scaffolds. After sintering, the linear shrinkage ratio was determined to be 31.6%. This method has been well-established as a mature synthetic technique in our research group [17,18,19]. Cubic SiC scaffolds were primarily fabricated in this work.

#### 2.2.2. Preparation of N-rGO Composites

N-rGO (denoted NC) composites were synthesized via a one-pot hydrothermal method. First, 0.036 g of GO was dispersed in 18 mL of deionized water by sonication (300 W, 40 kHz) for 1 h to yield a homogeneous brown dispersion (2 mg/mL). A total of 6 mL of ammonium hydroxide solution (28 wt% NH_4_OH) was subsequently introduced under magnetic stirring (500 rpm) for 1 h. The resulting mixture was transferred into a 50 mL Teflon-lined stainless steel autoclave and hydrothermally treated at 160 °C ± 5 °C for 12 h. The as-formed N-rGO hydrogel was thoroughly washed with deionized water (3~5 cycles), frozen at −60 °C for 24 h, and lyophilized (−50 °C, 0.1 mbar, 48 h) to afford the final product. Lyophilization was adopted to avoid capillary-induced graphene sheet restacking and porous structure collapse caused by conventional thermal drying. By removing water through sublimation without liquid phase formation, this method effectively preserves the three-dimensional porous architecture and exposed nitrogen-doped active sites of the N-rGO hydrogel. The overall synthetic yield of N-rGO is approximately 83%.

#### 2.2.3. Preparation of N-rGO@SiC Composites

N-rGO@SiC (denoted NC@SiC) was fabricated by introducing the SiC scaffold into the NC precursor suspension before hydrothermal treatment, followed by the identical hydrothermal reaction.

#### 2.2.4. Synthesis of N-rGO/S

N-rGO/S (denoted NGS) was synthesized by adding nano-sulfur to the NC precursor before ultrasonic dispersion, followed by hydrothermal treatment. Briefly, the GO dispersion was pre-sonicated for 0.5 h, after which 0.108 g of nano-sulfur powder (S: GO = 3:1) was added, and the mixture was sonicated for an additional 0.5 h before hydrothermal treatment. The overall synthetic yield of N-rGO/S is approximately 26%.

#### 2.2.5. Synthesis of N-rGO/S@SiC

N-rGO/S@SiC (denoted NGS@SiC) was synthesized via a similar route to NC@SiC, except using NGS instead of NC as the precursor.

### 2.3. Material Characterization

#### 2.3.1. Measurements and Characterizations

The crystal structure, microstructure, morphological features, chemical composition, and surface physicochemical properties of the as-prepared materials were systematically characterized via multiple analytical techniques. Detailed sample preparation protocols and instrument operating parameters for each characterization method are specified as follows.

##### Scanning Electron Microscopy (SEM) and Energy-Dispersive X-Ray Spectroscopy (EDS)

The surface microstructure and micromorphology of the samples were examined by field emission scanning electron microscopy (SEM). For sample preparation, the powdered sample was dispersed in anhydrous ethanol via ultrasonic treatment for 5 min to form a homogeneous dilute suspension. A small amount of the suspension was dropped onto a clean silicon wafer and dried naturally at room temperature. Subsequently, the dried sample was fixed on a conductive adhesive tape, and a thin layer of gold (Au) was sputtered on the sample surface for 60 s to enhance surface conductivity and eliminate charge accumulation during testing. The SEM images were acquired at an accelerating voltage of 10 kV with a working distance of 8~10 mm. Simultaneously, EDS integrated with the SEM instrument was utilized for localized elemental composition and distribution analysis. The EDS test was performed under the same accelerating voltage, with a collection time of 120 s, to accurately obtain the type, relative content, and spatial distribution of elements on the sample surface.

##### Transmission Electron Microscopy (TEM)

The fine microstructure, lattice structure, and internal morphological features of the materials were further observed by TEM. The TEM sample preparation procedure was as follows: a tiny amount of the powder sample was dispersed in 2 mL of anhydrous ethanol, and ultrasonic dispersion was performed for 10 min to achieve uniform dispersion and avoid particle agglomeration. A drop of the homogeneous suspension was carefully dripped onto a copper grid covered with a carbon film, and the excess liquid was absorbed with filter paper. The copper grid was placed in a dust-free drying oven and dried at 40 °C for 30 min to completely remove the solvent. TEM tests were conducted at an accelerating voltage of 200 kV, and high-resolution TEM (HRTEM) images were collected to analyze the lattice fringes, crystal defects, and microscopic structural characteristics of the samples.

##### X-Ray Diffraction (XRD)

Phase identification and crystal structure analysis of the samples were conducted by XRD. Prior to testing, the powder sample was fully ground into fine, uniform particles using an agate mortar, then evenly spread and compacted on a clean glass sample stage to form a flat and smooth test surface without obvious gaps or protrusions, ensuring no preferred orientation of the powder particles. The XRD measurement was performed on a conventional X-ray diffractometer with a Cu Kα radiation source (λ = 1.5406 Å), under a scanning 2θ range of 5~80°. The scanning speed was set to 5°/min with a step size of 0.02°, and the tube voltage and tube current were maintained at 40 kV and 40 mA, respectively. All tests were carried out at room temperature and atmospheric pressure to obtain accurate crystal phase diffraction patterns for phase matching and crystallinity analysis.

##### Raman Spectroscopy

Raman spectroscopy was employed to evaluate the reduction degree, structural defects, and molecular vibration characteristics of graphene oxide-based materials. For sample preparation, the dried powder sample was ground uniformly and placed flat on a clean glass slide to form a smooth test layer without surface unevenness. The Raman test was carried out on a confocal Raman microscope at room temperature. A 532 nm solid-state laser was used as the excitation source, with a laser power of 5 mW to prevent sample structural damage caused by excessive laser energy. The spectral scanning range was set from 100 cm to 4000 cm, the spectral resolution was 2 cm, and the integration time was 30 s with 3 cumulative scans to improve the signal-to-noise ratio. The obtained Raman spectra were used to analyze the characteristic peak intensity ratios, reflecting the defect density and reduction degree of graphene oxide in the composites.

##### X-Ray Photoelectron Spectroscopy (XPS)

XPS was used to analyze the surface elemental composition, chemical valence states, and bonding states of the samples. Before testing, the powder sample was pressed into a uniform thin sheet using a tablet press and fixed on the sample holder. The XPS measurement was performed in a high-vacuum test chamber (base pressure < 1 × 10 mbar) with an Al Kα monochromatic X-ray source (1486.6 eV). The survey spectrum was collected with a pass energy of 100 eV and a step size of 1 eV for full-spectrum elemental scanning. High-resolution narrow spectra of main elements (C, O, and other characteristic elements) were recorded with a pass energy of 20 eV and a step size of 0.1 eV for accurate fitting of chemical bonding states. All binding energy values were calibrated by the C 1s peak at 284.8 eV to eliminate test system errors.

##### N_2_ Sorption Measurements

The specific surface area, pore volume, and pore size distribution of the porous materials were measured via nitrogen adsorption–desorption (N_2_ sorption) isotherm tests. Detailed sample pretreatment and test parameters are described as follows: Prior to the adsorption test, approximately 100 mg of the dried sample was weighed and placed into a clean sample tube. The sample was degassed under vacuum conditions at 120 °C for 6 h to completely remove adsorbed water, residual ethanol, and other impurity gases on the sample surface and internal pores, ensuring the accuracy of pore structure data. After natural cooling to room temperature, the sample tube was transferred to the test station. The N_2_ sorption measurements were performed at a liquid nitrogen temperature of 77 K. The adsorption–desorption isotherms were recorded in the relative pressure (*P*/*P*_0_) range of 0.01~0.995. The Brunauer–Emmett–Teller (BET) model was applied to calculate the specific surface area of the sample, and the Barrett–Joyner–Halenda (BJH) model was used to analyze the mesopore size distribution and pore volume based on the desorption branch of the isotherm.

#### 2.3.2. Electrochemical Measurements

The as-synthesized composite was utilized as the working electrode without any binders and conductive additives, a platinum electrode as the counter electrode, and a mercury/mercury oxide (Hg/HgO) electrode as the reference electrode, with 6 mol/L KOH solution as the electrolyte. The area of the working electrode was approximately 0.9 cm^2^. To avoid overestimating the energy storage performance, the specific capacity was normalized by geometric electrode area. Since the actual active surface area is difficult to accurately measure, this method reduces experimental errors, conforms to practical application conditions, and ensures reliable and comparable data. A complete three-electrode electrochemical test system was thereby constructed for subsequent electrochemical performance characterization.

Electrochemical measurements were conducted in a three-electrode configuration using an electrochemical workstation. Cyclic voltammetry (CV) was performed between −1.0 and 0 V (vs. Hg/HgO) at scan rates of 5~100 mV/s. Electrochemical impedance spectroscopy (EIS) was recorded over 0.5 Hz~10 kHz. Galvanostatic charge–discharge (GCD) curves were collected at current densities of 1~10 mA/cm^2^ within the same potential window.

The areal specific capacitance (*C_GCD_*) was calculated from discharge curves using:

(1) $$C_{GCD}=\frac{A}{\nu \cdot \Delta V\cdot S}$$

where *C_GCD_* is the areal specific capacitance (F/cm^2^), *A* is the integral of the discharge curve (U-I curve), *v* is the discharge current (*A*), Δ*V* is the potential window (*V*), and *S* is the active material area (cm^2^).

## 3. Results and Discussion

### 3.1. Material Analysis of the Composite

**Figure 1:**
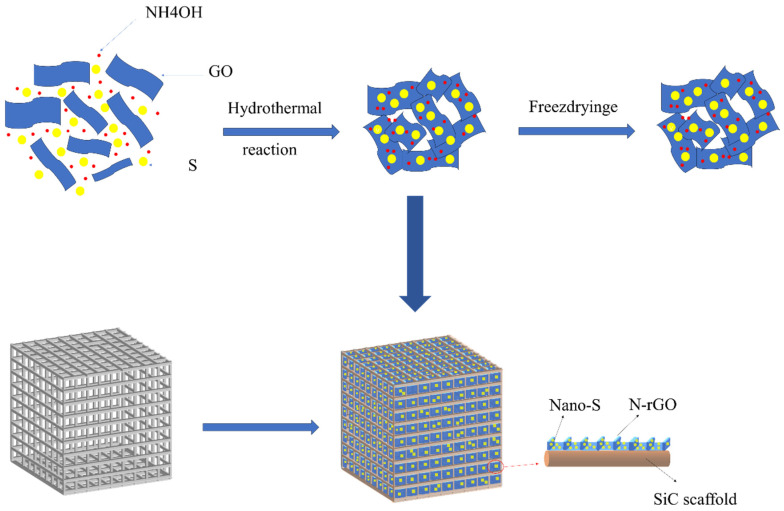
Synthesis of N-rGO/S@SiC.

The two-step preparation process illustrated in Figure 1 comprises (1) DLP 3D printing of porous SiC scaffolds, and (2) hydrothermal synthesis of the composites. Specifically, porous SiC scaffolds were fabricated via DLP photocuring followed by high-temperature sintering, following the method previously established by our group. During hydrothermal synthesis, GO undergoes self-assembly to fully infiltrate the SiC scaffold pores, thereby enhancing the electronic conductivity and energy storage capability of the composite. The incorporation of nano-S with GO promotes secondary pore formation, providing additional electron transport pathways, while the chemical bonding between sulfur and carbon contributes to improved structural stability. Furthermore, nitrogen doping enhances pseudocapacitive performance and electron transfer efficiency through electronic structure modulation, active site construction, and optimization of interfacial ionic transport [20,21,22]. Photographs and SEM images of the synthesis process are provided in Figure S2, confirming the successful self-assembly and attachment of GO onto the SiC surface. For brevity, N-rGO@SiC and N-rGO/S@SiC are hereafter denoted as NC@SiC and NGS@SiC, respectively.

**Figure 2:**
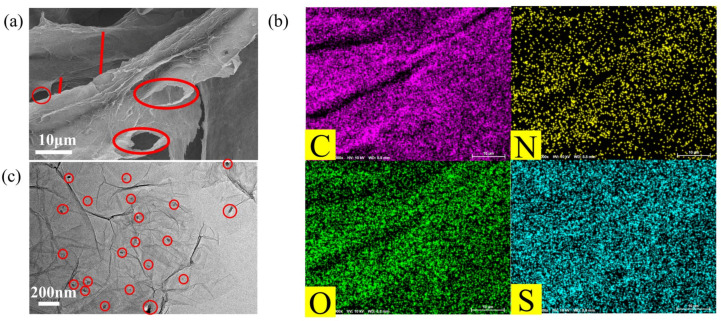
(**a**) SEM image of N-rGO/S. (**b**) TEM image of N-rGO/S. (**c**) EDS spectrum of N-rGO/S.

As shown in Figure 2a and Figure S3, the NGS composite exhibits a layered structure with large interlayer pores. The stacking and curling of these layers create an abundant three-dimensional interpenetrating network, while intralayer voids are also observed within the sheets. EDS mapping (Figure 2b) further verifies the homogeneous incorporation and uniform distribution of sulfur and nitrogen elements. Nano-S particles (marked by red circles in Figure 2c) are observed to be attached to the graphene surface. The size distribution of sulfur nanoparticles in NGS, statistically analyzed from 85 particles in Figure 2c and Figure S3, displays a Gaussian profile (Figure S4), with an average particle size of 16.90 nm ± 1.74 nm. This corroborates that nano-sized sulfur achieves improved dispersion within the graphene oxide matrix at the microscopic scale.

**Figure 3:**
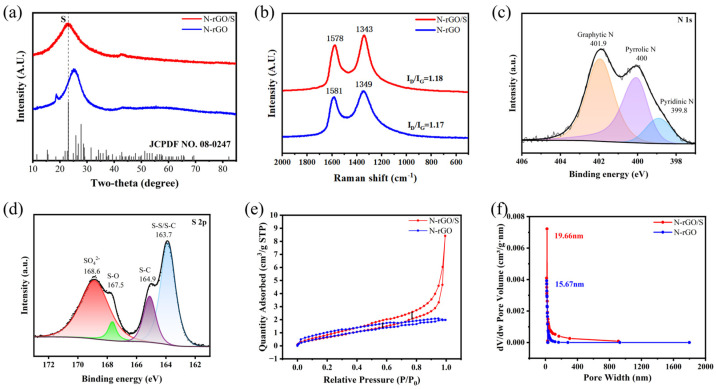
N-rGO and N-rGO/S (**a**) XRD. (**b**) Raman. (**c**) N 1s XPS spectrum of N-rGO/S. (**d**) N 2p XPS spectrum of N-rGO/S. (**e**) N_2_ adsorption–desorption isotherm. (**f**) BJH distribution diagram.

The structural features of the composites were investigated by X-ray diffraction (XRD). The patterns of NC, NGS, and sulfur are presented in Figure 3a. Two distinct diffraction peaks are exhibited by N-rGO, with the main peak at ~25.32° corresponding to the reflection of graphitic carbon [23]. Upon sulfur incorporation, only one prominent peak is observed in the NGS pattern, which is shifted to a lower angle (~22.3°) and broadened compared to N-rGO, consistent with the main peak of elemental sulfur (JCPDS No. 08-0247). The peak shift and broadening behaviors are primarily attributed to the structural regulation of the graphene skeleton induced by sulfur doping. Specifically, given that the atomic radius of sulfur is larger than that of carbon, sulfur atoms can be incorporated into the graphene lattice via lattice substitution or interlayer intercalation during the doping process, which enlarges the interlayer spacing of graphene sheets and thus results in the low-angle shift of the (002) diffraction peak. Furthermore, the differences in atomic size and electronegativity between sulfur and carbon atoms induce lattice distortion and micro internal stress, disrupt the ordered stacking structure of graphene layers, and introduce abundant lattice defects such as vacancies and topological defects. These defects effectively reduce the coherent scattering domain size of graphene crystals, which serves as the dominant reason for the obvious broadening of the (002) diffraction peak; meanwhile, the superposition effect of sulfur diffraction peaks further aggravates this peak broadening phenomenon. The above results confirm that sulfur atoms have been successfully doped into the skeleton of the composite material.

Further evidence of the doping and reduction process is provided by the Raman spectrum (Figure 3b). Two prominent peaks observed at 1365 and 1590 cm^−1^ are assigned to the characteristic D and G bands of carbon materials, respectively. The intensity ratio of the D band to the G band (I_D_/I_G_) is commonly used to evaluate the defect density of carbon materials, with a higher ratio indicating more defects and a higher reduction degree. Compared to N-rGO (I_D_/I_G_ = 1.17), the I_D_/I_G_ ratio of NGS increases to 1.18. The change in its value is related to the microstructural changes introduced after S doping in the composite material. The heteroatom doping induces plentiful structural defects within the graphene network, which expose sufficient electrochemically active sites and accelerate electrolyte ion transport, thereby improving the electrochemical capacitive properties [24,25].

The surface chemical composition and functional groups of the NGS composite were characterized by X-ray photoelectron spectroscopy (XPS), as shown in Figure 3c. Nitrogen analysis reveals three primary nitrogen species: pyridinic N (~398.8 eV), pyrrolic N (~400 eV), and graphitic N (~401.9 eV) [25,26]. The corresponding relative contents of the above three nitrogen configurations are 12.83 at.%, 39.01 at.%, and 48.16 at.%, respectively. Pyridinic N provides redox-active sites that directly contribute to Faradaic pseudocapacitance while enhancing electrolyte ion adsorption and interfacial charge transfer. Pyrrolic N is conducive to constructing electric double-layer/pseudocapacitive composite energy storage. Graphitic N is fully integrated into the carbon framework, significantly improving electron conduction and reducing charge transfer resistance, thereby providing efficient electron pathways for the Faradaic reactions of pyridinic N and pyrrolic N; synergistic effects yield optimal performance. As shown in Figure 3d, the S 2p spectrum of NGS comprises four peaks. Two peaks at 163.7 and 168.6 eV correspond to S–S/S–C bonds and SO_4_^2−^, respectively, indicating effective sulfur doping within graphene [22]. These strong sulfur-carbon interactions provide the driving force for the formation of the encapsulated structure. The sulfur content reaches 0.54 at.%, consistent with the SEM-EDS results.

Porosity characteristics were probed by N_2_ sorption analysis (Figure 3e). NC exhibits a Type II isotherm with low uptake and negligible hysteresis, indicative of tightly stacked graphene layers with limited mesoporosity. Conversely, NGS displays a Type IV isotherm featuring a pronounced H3 hysteresis loop at *P*/*P*_0_ = 0.4~0.9. The 4-fold increase in adsorption capacity for NGS relative to NC evidences that sulfur incorporation effectively mitigates graphene restacking and generates abundant mesopores. The steep uptake at *P*/*P*_0_ > 0.9 further signifies interparticle macropores, enabling rapid electrolyte ion transport. BJH analysis (Figure 3f) reveals a most probable pore diameter of 15.67 nm for NC with restricted pore volume, compared to 19.66 nm with substantially enhanced pore volume for NGS, corroborating mesopore dominance. These mesoporous architectures afford high specific surface area and shortened ion diffusion pathways, conducing to enhanced electrochemical performance [27,28].

These results confirm successful sulfur incorporation into graphene oxide. Subsequent introduction of SiC as a scaffold during synthesis yields NGS@SiC with robust integration within the SiC matrix. The results are displayed in Figure S5. The composite delivers a compressive strength of 93.5 MPa, while that of the NC self-assembled xerogel is merely 0.01 MPa. There exists a difference of four orders of magnitude in mechanical strength between them, which confirms that the SiC skeleton dominates the improvement of electrode mechanical properties.

### 3.2. Electrochemical Performance Testing and Analysis

**Figure 4:**
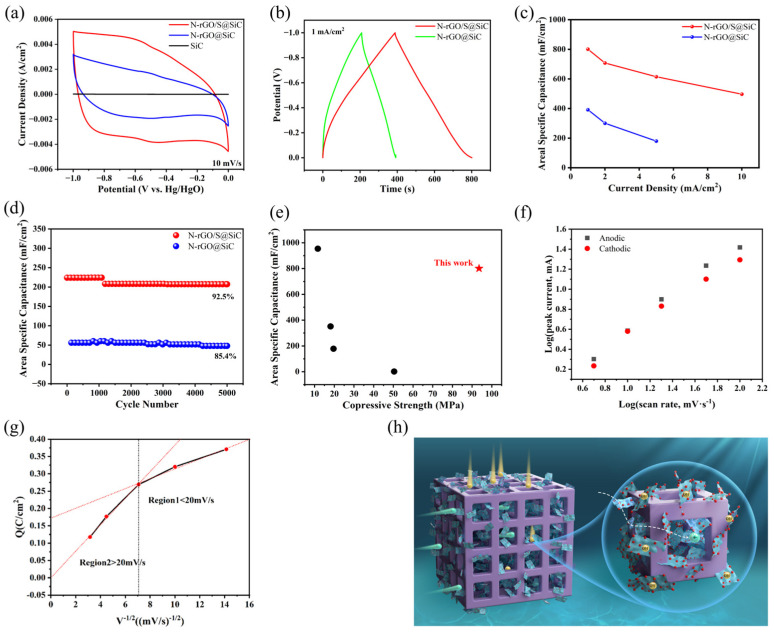
(**a**) CV curves of N-rGO@SiC and N-rGO/S@SiC at a scan rate of 10 mV/s. GCD curves of (**b**) N-rGO@SiC and N-rGO/S@SiC at a current density of 1 mA/cm^2^ and (**c**) Areal specific capacitance of N-rGO@SiC and N-rGO/S@SiC electrode at various current densities. (**d**) Long-cycle curve of N-rGO/S@SiC and N-rGO@SiC. (**e**) Compressive strength and areal specific capacitance comparison with as-reported electrode materials. (**f**) b value determination of normalized anodic and cathodic peak currents. (**g**) Plot of *Q* versus *v*^−1/2^ for N-rGO/S@SiC electrode. (**h**) Energy storage mechanism diagram of N-rGO/S@SiC composite.

The composite was evaluated as a supercapacitor electrode in a three-electrode configuration. The potential window was −1.0 to 0 V (vs. Hg/HgO). At a scan rate of 10 mV/s, pure SiC exhibited no capacitive characteristics due to its poor intrinsic conductivity (Figure 4a). In contrast, NGS@SiC displayed near-rectangular CV curves, demonstrating its energy storage mechanism combines electric double-layer capacitance and pseudocapacitance behavior. Broad redox peaks were also observed, attributed to the introduced nitrogen heteroatoms. The CV curve of NGS@SiC demonstrated a larger integrated area than that of NC@SiC, indicating higher specific capacitance. The increase in double-layer capacity reflected by the CV curve indicates the increase in micropores of the composite material after S doping. As shown in Figure S6, the CV curves of NGS@SiC at various scan rates maintain a nearly rectangular shape with increasing scan rate from 5 mV/s to 100 mV/s, indicating the favorable rate capability of the material. These results indicate that NGS@SiC primarily exhibits electric double-layer capacitance characteristics, accompanied by pseudocapacitance contributions from nitrogen functionalities [29,30].

GCD profiles at 1 mA/cm^2^ (Figure 4b) reveal approximately isosceles triangular shapes for both samples, characteristic of ideal electric double-layer capacitance with excellent reversibility [31]. Prolonged discharge duration is exhibited by NGS@SiC relative to NC@SiC, corroborating higher specific capacitance. The GCD curves at various current densities (Figure S7) maintain a nearly triangular shape even at 10 mA/cm^2^, demonstrating excellent rate capability. These results are consistent with CV analysis. The enhanced charge storage with sulfur incorporation is ascribed to (i) a richer mesoporous structure. This is mainly attributed to the secondary pore-making of S. The newly formed mesoporous structure is conducive to ion transfer and (ii) higher electrical conductivity. The Nyquist curve of NGS@SiC shows a smaller charge transfer resistance (R_ct_), mainly due to the chemical bond formed between sulfur and graphene, which enhances the electronic coupling at the interface (Figure S8). Meanwhile, the rich mesoporous structure shortens the ion diffusion path, jointly promoting the rapid charge transfer at the interface.

Areal specific capacitances as a function of current density are presented in Figure 4c. NGS@SiC delivers 800.7 mF/cm^2^ at 1 mA/cm^2^, retaining 76.7% at 5 mA/cm^2^—substantially exceeding NC@SiC (391.6 mF/cm^2^, 46.0%). The superior capacitance and rate performance stem from synergistic C, N, S effects and efficient charge storage in mesopores generated via secondary pore formation. Figure 4d indicates that after 5000 cycles, the specific capacitance of NGS@SiC decreased from 224.0 mF/cm^2^ to 207.2 mF/cm^2^, with a capacitance retention rate of 92.5%, which is significantly superior to that of NC@SiC (85.4%). This is mainly attributed to the strong interaction force between sulfur and graphene oxide, which enables the favorable growth of N-rGO/S within the porous SiC, thereby effectively improving the cycle stability of the electrode material.

As shown in Table S1, significant progress has been achieved in the field of structural supercapacitors in recent years through material structure design and interface optimization. Representative achievements include PCW (112 mF/cm^2^) [32], rGO/CuO (178.28 mF/cm^2^) [33], VG/MnO_2_ (30.7 mF/cm^2^) [34], CNTs/PPy (129.13 mF/cm^2^) [35], and C800-PANI-LGE (162 mF/cm^2^) [14], ZnO/CA (295F/g) [36] In comparison, the areal specific capacitance of NGS@SiC is at a relatively high level.

The NGS@SiC composite material prepared in this work exhibits outstanding electrochemical and mechanical performance, achieving an areal specific capacitance of 800.7 mF/cm^2^ and a compressive strength of 93.5 MPa, significantly surpassing existing carbon fiber-modified structural capacitors. Compared to previously reported fiber-reinforced polymer-based structural supercapacitors (Figure 4e): carbon fiber/glass fiber-reinforced (1.16 mF/cm^2^, 50.4 MPa) [37], fiber-carbon black cement-based (953.9 mF/cm^2^, 11.6 MPa) [38], PCSE (351 mF/cm^2^, 18.1 MPa) [39], and PC0.6 (178.28 mF/cm^2^, 19.6 MPa) [40]. Notably, the NGS@SiC material synergistically optimizes both metrics, overcoming the typical trade-off between mechanical strength and energy storage capacity.

### 3.3. Analysis of Energy Storage Mechanism and Electrochemical Active Surface Area

It has been proven that N, S-codoped carbon materials exhibit two typical energy storage mechanisms, namely electric double-layer ion adsorption and faradaic redox reaction induced by heteroatom doping. To clarify the underlying mechanism for the superior capacitive performance of NGS@SiC, the charge storage kinetic characteristics were systematically explored in this work. The power-law equation was adopted to distinguish whether the electrochemical energy storage process is governed by solid-state ion diffusion or surface capacitive behavior [41,42]:

(2) $$i=av^{b}$$

where *v* is the scan rate (mV/s), *i* is the peak current density (mA/cm^2^), and *a* and *b* are adjustable parameters. The *b*-value, ranging from 0.5 to 1, distinguishes between diffusion-controlled (0.5) and capacitive-dominated (1) charge storage mechanisms. The *b* values were measured at −0.6 V potential.

To probe the fundamental charge storage behavior of the NGS@SiC electrode, log(*i*) versus log(*v*) plots were constructed for anodic and cathodic peaks at scan rates of 5~100 mV/s (Figure 4e). The *b*-values for anodic and cathodic peaks were determined to be 0.71 and 0.77, respectively, indicating a charge storage mechanism dominated by electric double-layer capacitance with pseudocapacitive contributions. At scan rates below 20 mV/s, *b*-values approach unity for both peaks, signifying surface-controlled kinetics. At elevated scan rates (>20 mV/s), b-values decrease to 0.75 (cathodic) and 0.66 (anodic), attributable to increased ohmic resistance and diffusion limitations.

To further quantitatively separate different charge storage components, the Trasatti analytical method was employed for in-depth mechanism research [43,44]:

(3) $$Q=Q_{c}+Q_{d}$$

where *Q* is the total stored charge, *Q_c_* is the charge related to the capacitive process, and *Q_d_* is derived from the diffusion behavior. Assuming that semi-infinite diffusion is involved in the charge-discharge process, the contributions of different kinetic processes can be obtained by extrapolating the relationship curve between *Q* and *v*^−1/2^, as shown in Figure 4g. The scan rates are divided into two regions: Region 1 (*v* < 20 mV/s) is the capacitive control region, and Region 2 (*v* > 20 mV/s) is the diffusion control region. In Region 1, the change of charge *Q* with scan rate is not obvious; extrapolating the curve to the vertical axis (*v*^−1/2^ = 0), the obtained intercept is the charge contributed by the pure capacitive behavior, and *Q_c_* is calculated to be 0.1722 C/cm^2^. In Region 2, *Q* decreases approximately linearly with the decrease of *v*^−1/2^ (i.e., the increase of scan rate). Extrapolating the curve of Region 2 to the vertical axis, the charge contributed by diffusion, *Q_d_*, is obtained as 0.0001 C/cm^2^. The above results indicate that the charge storage kinetics of the NGS@SiC composite is dominated by capacitive behavior, which is also the reason for its fast reaction kinetics.

The anodic and cathodic current densities were collected at a fixed potential of −0.6 V from CV curves measured at different scan rates (10, 20, 50 mV/s). The average double-layer current density was calculated by

(4) $$j_{dl}=\frac{j_{a}-j_{c}}{2}$$

The double-layer capacitance *C_dl_* was obtained from the linear fitting slope between *j_dl_* and scan rate, and the relevant fitting diagram is presented in Figure S9. The electrochemically active surface area (*ECSA*) was further calculated via

(5) $$ECSA=\frac{C_{dl}}{C_{s}}$$

where the standard specific capacitance *C_s_* was set as 0.04 mF/cm^2^ for the aqueous electrolyte system. The calculated *ECSA* value is 4.35 cm^2^. Combined with the above Trasatti analysis results, the relatively large electrochemically active surface area provides abundant accessible active sites, which greatly facilitate surface-controlled capacitive behaviors and account for the dominant capacitive charge storage characteristic of NGS@SiC composites.

The energy storage mechanism of the composite is illustrated in Figure 4h. Electrons are transported through the rGO coating on the SiC skeleton, ensuring high electronic conductivity, while ions migrate from the electrolyte into the active material via abundant pores, guaranteeing high ionic conductivity. The mesoporous structure greatly shortens the ion diffusion distance. Meanwhile, nitrogen doping, sulfur doping, porous structure, and enhanced conductivity work in synergy to jointly improve the overall electrochemical performance. The inherent capacitive-dominated energy storage characteristic further enables the composite to possess excellent rate capability.

## 4. Conclusions

In summary, this study addresses the challenge of balancing mechanical properties and energy storage in structural capacitors by developing a composite featuring a high-strength porous SiC scaffold infiltrated with the high-performance electrode material N-rGO/S. This architecture facilitates both electron transfer and ion transport. Simultaneously, the introduction of sulfur enables secondary pore formation, increasing mesopore volume density. The resulting C–S bonds enhance structural stability and further boost specific capacitance. The composite exhibits a compressive strength of 93.5 MPa, an areal capacitance of 800.7 mF/cm^2^@1 mA/cm^2^, and 92.5% capacitance retention after 5000 cycles. These results demonstrate that this material is an excellent structure-energy storage dual-functional material. This novel composite offers a new approach for constructing structure-storage dual-functional materials through the efficient integration of porous high-strength scaffolds with active materials.

## Data Availability

The original contributions presented in this study are included in the article. Further inquiries can be directed to the corresponding author.

## Supplementary Materials

The following supporting information can be downloaded at: https://www.mdpi.com/article/10.3390/nano16110656/s1, Figure S1: Hydrolysis and condensation mechanism for the preparation of MK-TMSPM; Figure S2: (a) Optical photograph of the 3D printed SiC scaffold coated with NC. (b) NC growth on the outer surface of the SiC scaffold. (c) SEM image of NC filling within the SiC scaffold. (d) SEM image of NC grown on the SiC scaffold; Figure S3: TEM images of N-rGO/S at different positions: (a,b); Figure S4: The size distribution histogram of S; Figure S5: Force–deformation curves of N-rGO/S@SiC and N-rGO (b) Column chart of compressive strength for N-rGO/S@SiC and N-rGO; Figure S6: CV curves of N-rGO/S@SiC at different scan rates; Figure S7: Charge-discharge curves of N-rGO/S@SiC at various current densities; Figure S8: Nyquist plots of N-rGO/S@SiC and N-rGO@SiC; Figure S9: Linear fitting of the double-layer current density (*j_dl_*) versus scan rate (*v*) for NGS@SiC; Table S1: Performance comparison with reported supercapacitor electrodes.
