# Supplementary material for: N-rGO/S@porous SiC Composite with Multidimensional Hybrid Architectures for Structural Energy-Storing Applications
Source: Nanomaterials (Basel). 2026 May 23;16(11):656. doi: 10.3390/nano16110656 (PMC13258452; doi:10.3390/nano16110656)
Supplement: Supplementary file 1 [file nanomaterials-16-00656-s001.zip › nanomaterials-4331246-supplementary.pdf]

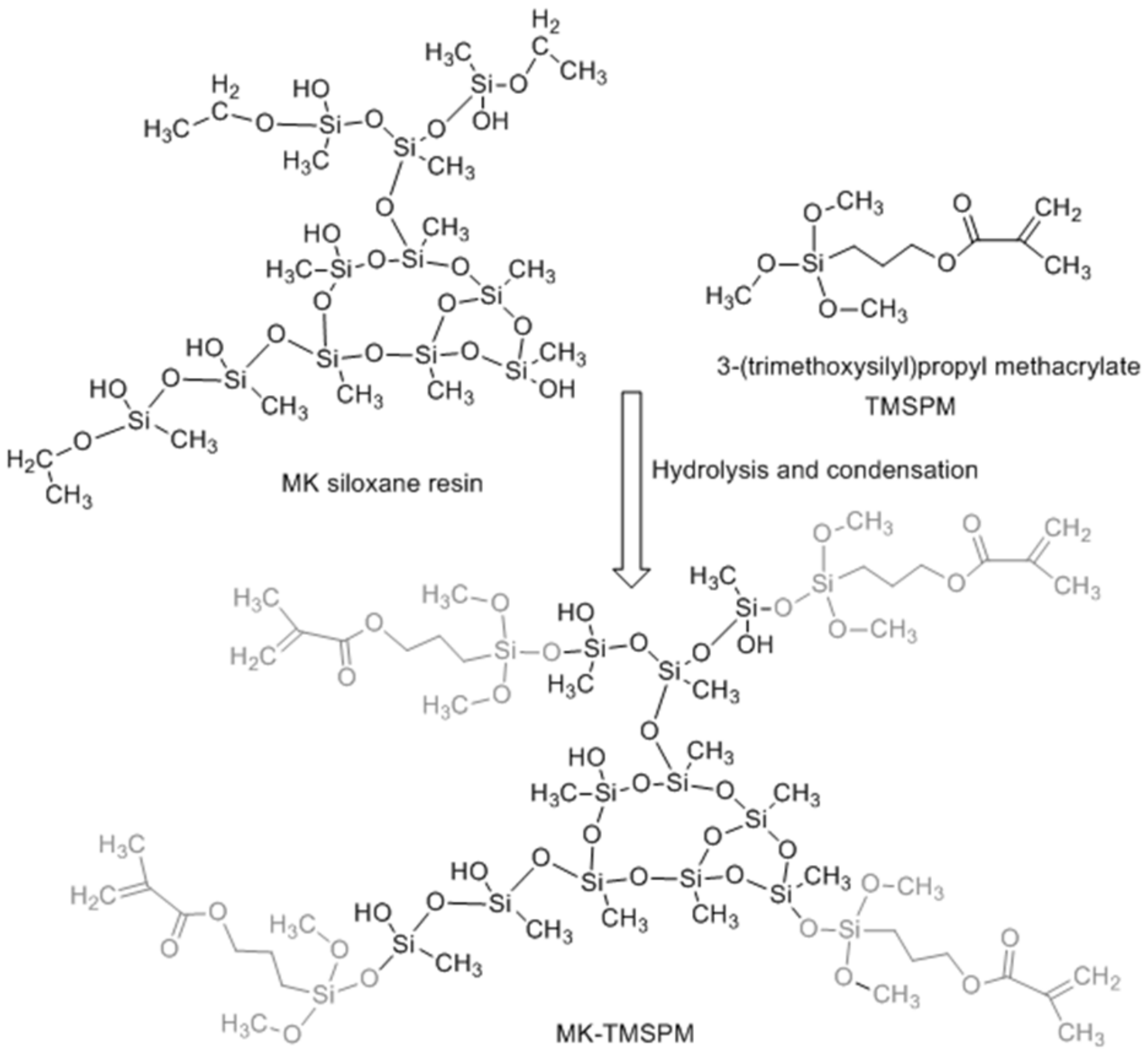

Figure S1. Hydrolysis and condensation mechanism for the preparation of MK-TMSPM.

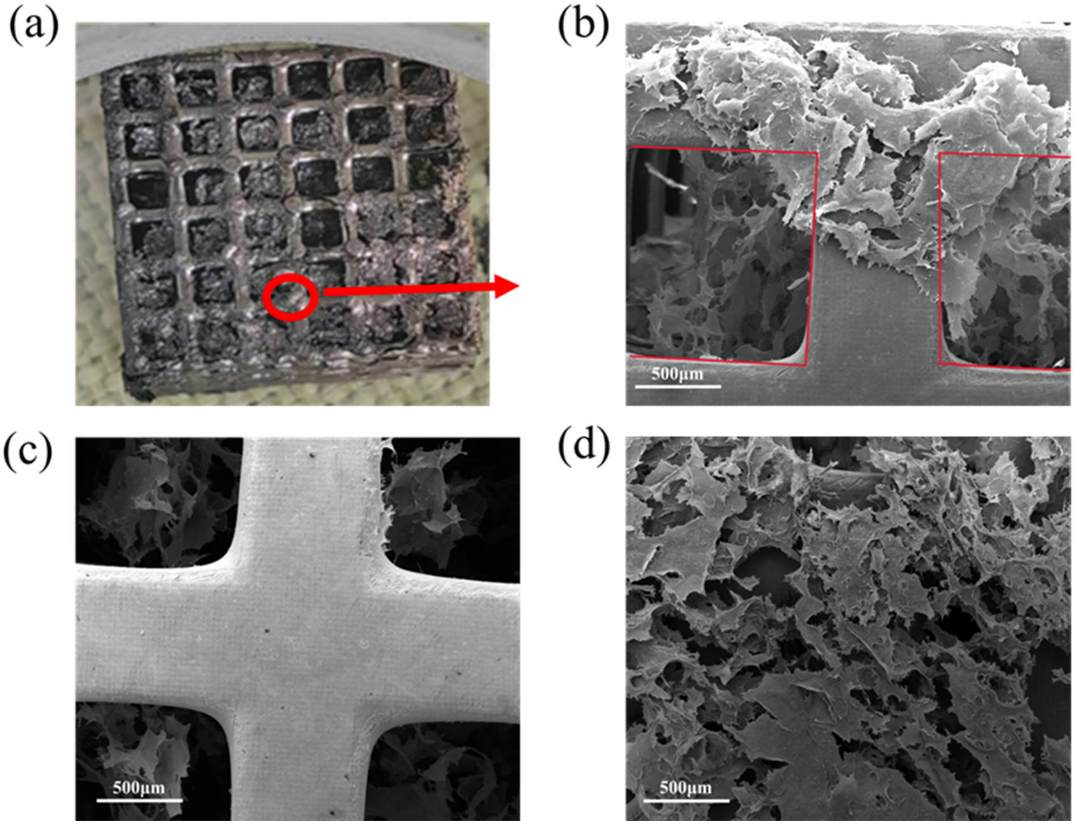

Figure S2. (a) Optical photograph of the 3D printed SiC scaffold coated with NC. (b) NC growth on the outer surface of the SiC scaffold. (c) SEM image of NC filling within the SiC scaffold. (d) SEM image of NC grown on the SiC scaffold.

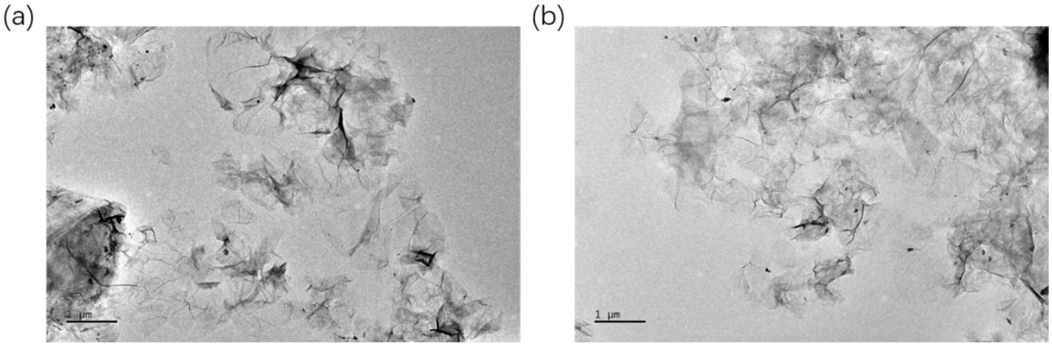

Figure S3. TEM images of N-rGO/S at different positions: (a) and (b).

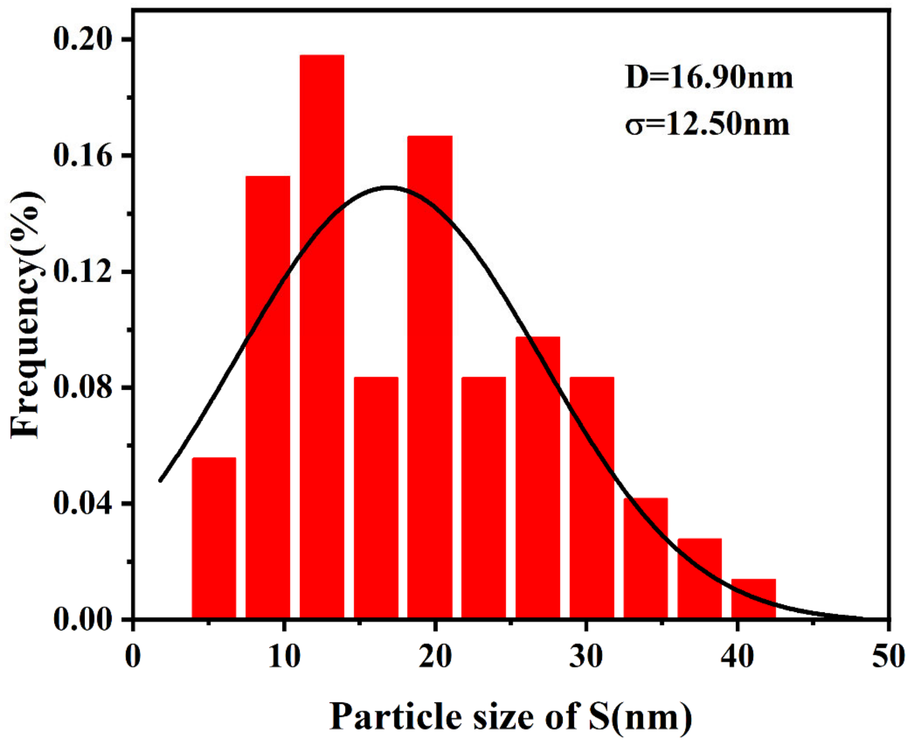

Figure S4. The size distribution histogram of S.

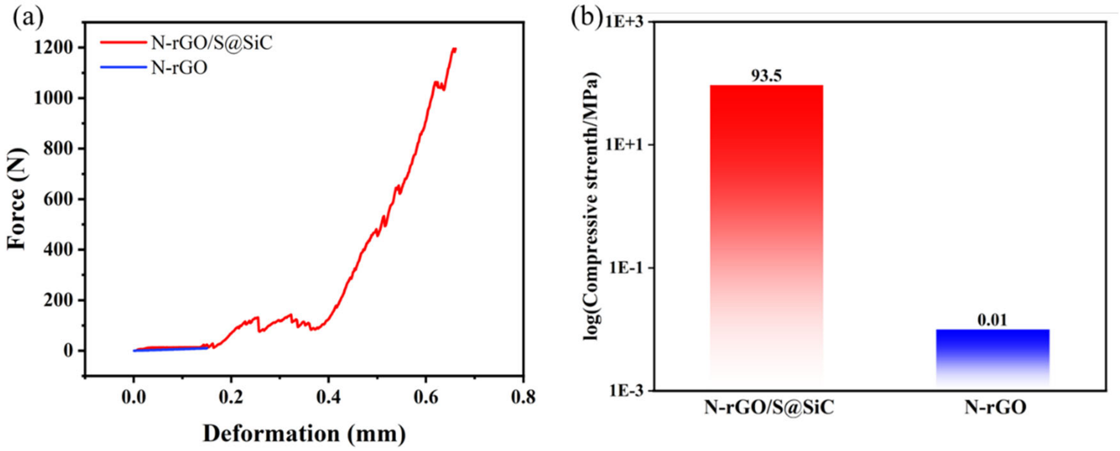

Figure S5. (a) Force–deformation curves of N-rGO/S@SiC and N-rGO (b) Column chart of compressive strength for N-rGO/S@SiC and N-rGO.

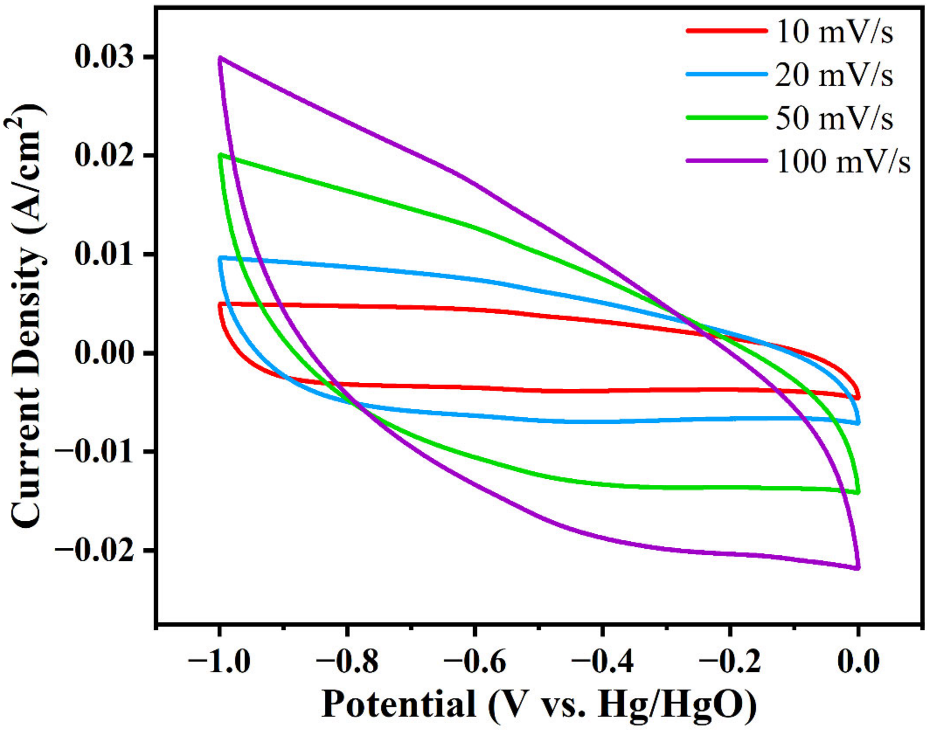

Figure S6. CV curves of N-rGO/S@SiC at different scan rates.

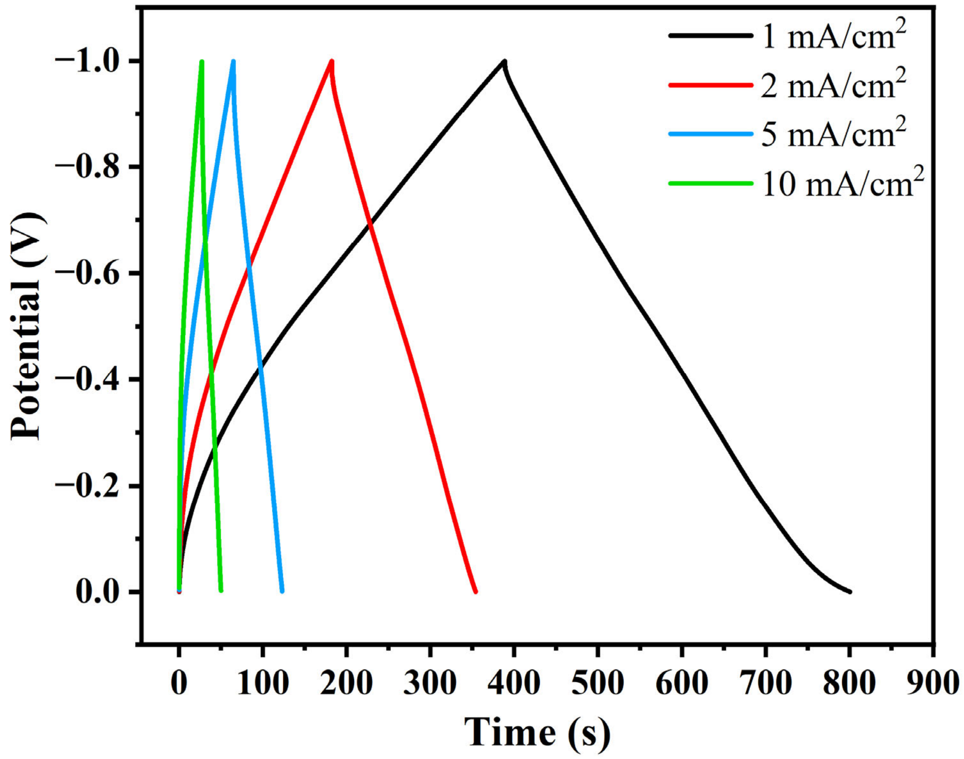

Figure S7. Charge-discharge curves of N-rGO/S@SiC at various current densities.

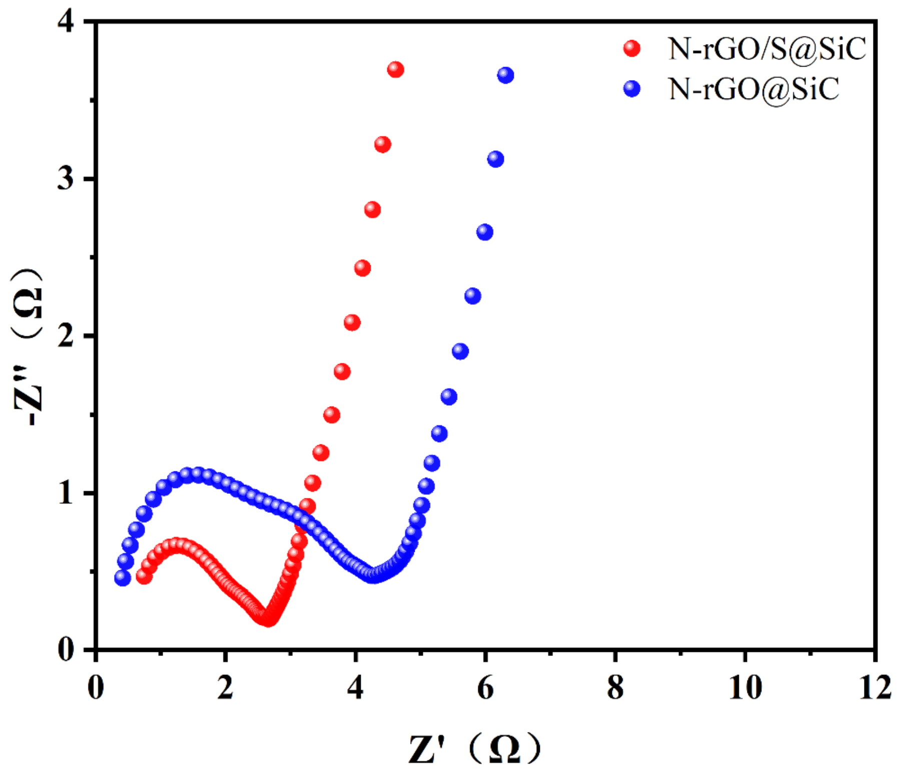

Figure S8. Nyquist plots of N-rGO/S@SiC and N-rGO@SiC.

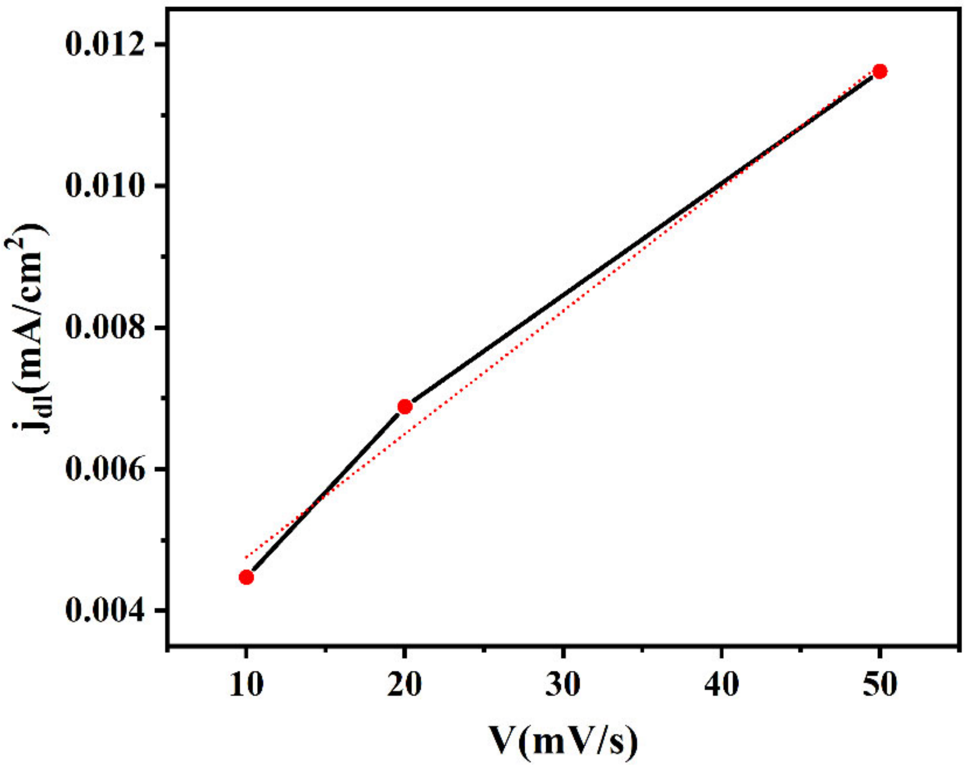

Figure S9. Linear fitting of the double-layer current density (j_{dl}) versus scan rate (v) for NGS@SiC.

Table S1. Performance comparison with reported supercapacitor electrodes.

Electrode material	Electrolyte	Voltage window	specific capacitance	Test configuration
SiC/rGO/S	Alkaline liquid	-1-0 V	800.7	three-electrode configuration
Delignified wood/MXene/Polypyrrole	Cellulose-based gel electrolyte	1.2-2.0 V	112 mF/cm^2	two-electrode configuration
Carbon fiber/rGO/CuO	PAA-cement composite solid electrolyte	0–4.0 V	178.28 mF/cm^2	two-electrode configuration
Carbon fiber/vertical graphene/MnO_2	Polymer solid electrolyte	0–2.0 V	30.7 mF/cm^2	two-electrode configuration
Carbon nanotube fiber/CNTs-PPy	Gel electrolyte	0–1.6 V	129.13 mF/cm^2	two-electrode configuration
rGO/MnO_x/carbon nanotube	Gel electrolyte	0–2.0 V	162 mF/cm^2	two-electrode configuration
Transition metal oxide/carbon aerogel	Alkaline liquid electrolyte	0–1.0 V	295 F/g	three-electrode configuration

Note: The specific capacitances of this work were measured via a three-electrode system, while some reference data were obtained from two-electrode devices, which may lead to differences in test conditions.
